# Infrared Coagulation: A Treatment Option for Cervical Intraepithelial Neoplasia 

**DOI:** 10.1200/JGO.17.00038

**Published:** 2017-08-31

**Authors:** Pantelis Vassiliu, Christos Bartsokas

**Affiliations:** **All authors**: Attikon University Hospital, Athens, Greece

## TO THE EDITOR:

In their recent article in *Journal of Global Oncology*, after meticulous evaluation of current literature and experience, Jeronimo et al^1^ present guidelines for secondary prevention of cervical cancer. Among those is the recommended treatment of grade 3 or greater cervical intraepithelial neoplasia (CIN) lesions with loop electrosurgical excision procedure (LEEP). Although LEEP is a common, well-studied, and effective procedure, it has complications, including cervical bleeding, stenosis, and pregnancy-related adverse outcomes.^2,3^ Thus, bridging knowledge from anal surgery, we propose the use of infrared coagulation (IRC) as a treatment option for CIN lesions to diminish or even eliminate complications. IRC effectively ablates anal lesions in an area (anus) with denser sensory innervation and microbial load than the cervix and in an area that experiences daily functional tissue stretching as a result of normal defecation, which does not occur in the cervical area. As a result of all these negative characteristics of the anal area, there have been no attempts to use LEEP in the anus because severe morbidity would result. However, using the friendly energy of IRC, anal dysplasia is treated effectively, especially when anal intraepithelial neoplasia covers a limited surface of the anal canal epithelium. This result is attributed to the fact that IRC penetrates fully the epithelium,^4^ which is the locum of intraepithelial lesions, leaving deeper tissues unaffected, which is not the case with LEEP.

On the basis of biologic similarities between anal and cervical dysplasia, the causal relationship of human papillomavirus with both lesions, and the similar depth of the epithelium in each of the two anatomic areas, successful treatment of anal intraepithelial lesions with IRC could be applied to treating cervical lesions^5-8^ with fewer adverse events compared with LEEP.

We recognize the key differences in anal and cervical screening,^9^ but given the aforementioned similarities between CIN and anal intraepithelial lesions, it seems that IRC could stand as an alternative treatment^10^ in selected patients with CIN and is worthy of a thorough scientific comparison against the currently recommended treatment of LEEP.
